# Knee arthrodesis versus above-the-knee amputation after septic failure of revision total knee arthroplasty: comparison of functional outcome and complication rates

**DOI:** 10.1186/s12891-017-1806-8

**Published:** 2017-11-13

**Authors:** Sven Hungerer, Martin Kiechle, Christian von Rüden, Matthias Militz, Knut Beitzel, Mario Morgenstern

**Affiliations:** 1 BG Unfallklinik Murnau, Prof. Küntscher Str. 8, Murnau, 82418 Germany; 2 Institute of Biomechanics, Paracelsus Medical University Salzburg and BG Unfallklinik Murnau, Prof. Küntscher Str. 8, Murnau, 82418 Germany; 3 Department of Orthopedic Sports Medicine, Technische Universität München, Isamningerstr. 22, 81675 Munich, Germany; 4 Department of Orthopaedic Surgery and Traumatology, University Hospital Basel, Spitalstr. 21, 4031 Basel, Switzerland

**Keywords:** Prosthetic joint infection, Revision total knee arthroplasty, Knee-arthrodesis, Above-the-knee amputation

## Abstract

**Background:**

After septic failure of total knee arthroplasty (TKA) and multiple revision operations resulting in impaired function, bone and/or soft-tissue damage a reconstruction with a revision arthroplasty might be impossible. Salvage procedures to regain mobility and quality of life are an above-the-knee amputation or knee arthrodesis. The decision process for the patient and surgeon is difficult and data comparing arthrodesis versus amputation in terms of function and quality of life are scarce. The purpose of this study was to analyse and compare the specific complications, functional outcome and quality of life of above-the-knee amputation (AKA) and modular knee-arthrodesis (MKA) after septic failure of total knee arthroplasty.

**Methods:**

Eighty-one patients treated with MKA and 32 patients treated with AKA after septic failure of TKA between 2003 and 2012 were included in this cohort study. Demographic data, comorbidities, pathogens and complications such as re-infection, implant-failure or revision surgeries were recorded in 55MKA and 20AKA patients. Functional outcome with use of the Lower-Extremity-Functional-Score (LEFS) and the patients reported general health status (SF-12-questionnaire) was recorded after a mean interval of 55 months.

**Results:**

A major complication occurred in more than one-third of the cases after MKA and AKA, whereas recurrence of infection was with 22% after MKA and 35% after AKA the most common complication. Patients with AKA and MKA showed a comparable functional outcome with a mean LEFS score of 37 and 28 respectively (*p* = 0.181). Correspondingly, a comparable physical quality of life with a mean physical SF-12 of 36 for AKA patients and a mean score of 30 for MKA patients was observed (*p* = 0.080). Notably, ten AKA patients that could be fitted with a microprocessor-controlled-knee-joint demonstrated with a mean LEFS of 56 a significantly better functional outcome than other amputee patients (*p* < 0.01) or MKA patients (*p* < 0.01).

**Conclusion:**

Naturally, the decision process for the treatment of desolate situations of septic failures following revision knee arthroplasty is depending on various factors. Nevertheless, the amputation should be considered as an option in patients with a good physical and mental condition.

## Background

Prosthetic joint infections (PJI) following total knee arthroplasty (TKA) pose a devastating complication, since eradication of infection and restoration of functionality present a significant challenge to both patients and surgeons [1, 2]. Despite tremendous efforts and targeted therapy, infection reoccurs in up to 14 to 28% after revision TKA and causes severe morbidity as well as substantial treatment costs [3, 4]. If infection cannot be eradicated or if multiple revision TKAs led to loss of soft-tissue, extreme bone defects or instability as well as deficiency of the extensor apparatus successful reconstruction or control of infection using revision TKA may no longer be possible [5, 6]. In these cases knee-arthrodesis or above-the-knee amputation (AKA) are beside resection arthroplasty often the only treatment options [2, 5]. Wu et al. performed a systematic review on treatment options in persistent infection after failed revision TKA and concluded that arthrodesis should strongly be considered in this case to control infection and to maximize function [7]. In contrast, Rohner et al. recently reported an infection persistence of 50%, substantially impaired quality of life and pain after knee-arthrodesis. They concluded that bone fusion following septic failure of revision TKA should be regarded with scepticism [2]. On the other hand, poor functional outcome and high complication rates of more than 30% are also described for AKA after TKA [8–10]. There are scant data on directly comparing functionality and complication rates of AKA and knee-arthrodesis performed after septic failure of TKA. Solely, one retrospective study compared the functional outcome of bone fusion and AKA after PJI in small numbers [11]. There is no study comparing AKA with access to modern orthotics and modular knee arthrodesis (MKA) in this situation. Knee-arthrodesis with modular endoprosthesis provides advantages over bone fusion including immediate fixation and weight bearing as well as modularity, which allows the reconstruction of segmental deficits [12].

Therefore the central aims of our study was to analyse the clinical course, complications, functionality and quality of life of AKA and MKA after septic failure of TKA. We hypothesize that neither AKA nor MKA after septic failure of TKA is superior in terms of functional outcome and complication rates and that the treatment decision process should be judged individually according to the patients` overall condition and the local bone and soft-tissue status.

## Methods

All patients treated in our department over a ten-years time period (2003–2012) with MKA or AKA after septic failure of revision TKA were included in this retrospective cohort study. Additional inclusion criteria were: minimum follow-up interval of 12-month, a sufficient patient data set and complete radiographic imaging studies. A PJI was diagnosed according to the American Academy of Orthopaedic Surgeons clinical practice guideline [13].

Demographic data was collected and the overall medical condition of the patient was evaluated using the Charlson comorbidity index (CCI) [14]. The initial infecting pathogens detected in the underlying PJI were documented. Patients were seen in regular visits (minimum visits after surgery: 6 weeks, 6 months, 1 year) and underwent physical and radiographic examination.

In patients with KA the following items were documented: implant positioning and leg length discrepancy. Distance arthrodesis was performed with a modular system (Peter Brehm GmbH, Weisendorf, Germany). Major complications after arthrodesis such as re-infection, implant-failure /−loosening or fracture were documented and surgical revisions like implant exchange, debridement or amputation were quoted. A recurrence or persistent infection was defined when local and/ or systemic signs of infection or one of the mentioned diagnosis criteria for PJI were present [13]. Loosening was defined as migration of the implant and the presence of a radiolucent liner larger than 2 mm [2]. Survival of the implant or arthrodesis was deemed to be the absence of above-mentioned complications or surgical revisions.

In patients with AKA the following was documented: level of amputation, fitted with a functional prosthesis and type of prosthesis (mechanic and microprocessor-controlled-knee-joint). Major complications after amputation such as stump healing disorder, recurrence of infection and revision amputation were recorded.

In 58 patients functional outcome was assessed with use of the Lower Extremity Functional Scale (LEFS) [15] and the SF-12 [16]. According to previous validation studies the SF-12 is comparable to the SF-36 for assessing patients` physical (Physical Component Summary; PCS) and emotional quality of life (Mental Component summary; MCS) [17]. The LEFS describes the functionality of the lower extremity (maximum score of 80 equates to the best functional outcome) [15]. Patients, in which amputation was performed after MKA, were excluded from analysis of functional outcome.

### Statistical analysis

Statistical analysis was performed using SPSS® Statistics for Windows 19.0 (IBM Corp., Armonk, New York, U.S.A.). Results in this study are presented as mean values with standard deviation. Significance for categorical data was calculated using the Pearson’s chi-squared test. Analysis of variance was used to detect differences between the groups. Numeric data were tested for normal distribution with the Kolmogorov Smirnov Test. Assuming parametric data, statistical differences were tested using the paired T-test for independent variables. A result was considered to be statistically significant with *p*-value <0.05. Implant survival was calculated by Kaplan-Meier survival plot. The functional outcome, assessed with the LEFS was defined the primary aim. The complication rate, as well as the patients` physical (PCS) and emotional quality of life (MCS) were defined secondary aims.

## Results

### Overview – Patient cohorts

**Fig. 1:**
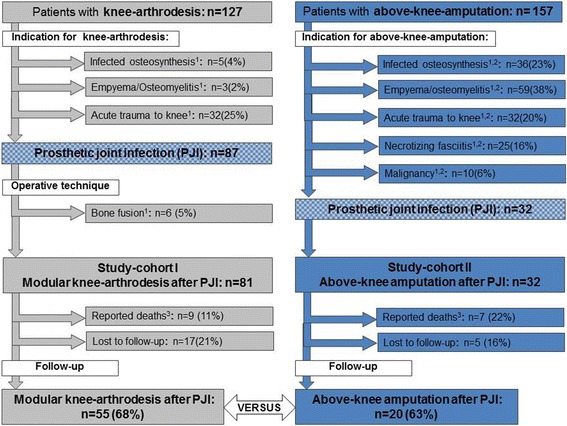
Graphic delineation of study cohorts; Footnotes: ^1^Exclusion criteria; ^2^Multiple indications possible; ^3^Within first postoperative year

In the time period between 2003 and 2012 we treated 127 patients with knee-arthrodesis and 157 patients with AKA due to various indications. Patients undergoing knee-arthrodesis or AKA due to other indication than PJI were excluded from this study. In total in 32 patients AKA and in 81 MKA was performed after septic failure of revision TKA and therefore patients were included in the current study. In six patients knee-arthrodesis after PJI was performed by bone fusion using an external fixator, plates or an intramedullary nail and they were excluded from this investigation (Fig. 1).

### Modular knee arthrodesis – Clinical course and complications

Demographic data and infection characteristics of MKA patients are summarized in Table 1. After MKA, three patients (4%) died postoperatively and death was related to the underlying infection or a serious postoperative complication. Six patients reportedly died in the first year after arthrodesis. In total, 17 patients with MKA were lost to follow-up examination, leaving 55 patients for analysis.

**Table 1:** Modular knee-arthrodesis (MKA) after prosthetic joint infection (PJI): demographic and clinical data, implant survival rate

Characteristic	MKA after PJI
Number of patients, n	81
Demographic data	
Mean age (in years), mean (sd; Min - Max)	68.6 (11.2; 29–85)
Male sex, n (%)	43 (53.1)
Charlson Comorbidity Index, mean (sd)	4.8 (2.0)
Death within 1st year, n (%)	9 (11.1)
Lost to follow-up, n (%)	17 (21.0)
Patients with min. Follow-up, n (%)	55 (67.9)
Disease causing pathogens^a^	
*S. aureus*, n (%)	25 (30.9)
*S. epidermidis*, n (%)	31 (38.3)
Others^2^, n (%)	25 (30.9)
Leg length discrepancy after MKA in cm, mean (sd)	1.8 (1.4)
Survival rate (SR) for MKA after PJI	
One – year SR	85.6%
Five – years SR	71.1%
Ten – years SR	60.9%

^a^Disease causing pathogen isolated in PJI leading MKA

During the follow-up period loosening occurred after eight (15%) arthrodeses. A peri-implant fracture was seen in four patients (7%) and a technical implant failure in one patient (2%). Re-infection was observed in 12 cases (22%). An amputation had to be performed due to persisting or recurrent infection in six patients (11%) (Table 2). In nine patients at least one re-arthrodesis had to be performed due to periprosthetic fracture, implant loosening or re-infection. This was leading to a total number of 93 arthrodeses in 81 patients. An overall survival rate for all 93 modular arthrodeses was after one year 86%, after five years 71% and after ten years 61% (Table 1).

**Table 2:** Modular knee-arthrodesis (MKA) versus Above-the-knee amputation (AKA) after prosthetic joint infection (PJI): clinical course, complications, functional outcome and quality of life

Characteristic	MKA after PJI	*p*-value	AKA after PJI
Patients with min. Follow-up (12 month), n (%)	55 (67.9)		20 (62.5)
Follow-up interval in month			
• Mean (sd)	53 (26)		62 (40)
• Min-Max	12–119		12–112
Complications			
Patients with major complication, n (%)	20 (36.4)	0.91	7 (35.0)
Recurrence of infection, n (%)	12 (21.8)	0.25	7 (35.0)
(Re-) Amputation, n (%)	6 (10.9)	0.31	4 (20.0)
MKA Loosening, n (%)	8 (14.5)	n/a	–
MKA Implant failure, n (%)	1 (1.8)	n/a	–
Peri-implant fracture, n (%)	4 (7.3)	n/a	–
Patients with functional follow up, n (%)	48 (59.3%)		10 (31.3%)
Functional follow-up examination			
LEFS^a^, mean (sd)	28 (13.7)	0.181	37 (26.4)
Physical SF-12, mean (PCS) (sd)	30 (9.1)	0.080	36 (14.5)
Mental SF-12, mean (MCS) (sd)	46 (11.2)	0.755	47 (12.0)

^a^LEFS = Lower Extremity Functional Scale. A maximum score of 80 equates to the best functional outcome

### Above-the-knee amputations –clinical course and complications

Demographic data and infection characteristics of amputee patients are summarized in Table 3. After AKA four patients (13%) died postoperatively and death was related to the underlying disease or a serious postoperative complication. Two patients reportedly died in the first year after in AKA. In total, five patients (16%) with AKA were lost to follow-up examination, leaving 20 patients for analysis (Table 3). At follow-up 80% of the patients were fitted with a functional prosthesis (*n* = 16), six of them with a mechanic knee joint (30%) and ten with a microprocessor-controlled-knee-joint (50%). After initial AKA a revision surgery with irrigation and debridement was required due to non-healing stumps or recurrent infections in seven patients (35%). Re-amputation had to be performed in four cases (20%) (Table 2).

**Table 3:** Above-the-knee amputation (AKA) after prosthetic joint infection (PJI): demographic and clinical data, level of AKA and orthotics

Characteristic	AKA after PJI
Number of patients, n	32
Demographic data	
Mean age (in years), mean (sd; Min – Max)	63.4 (14.4; 29–85)
Male sex, n (%)	17 (53.1)
Charlson Comorbidity Index, mean (sd)	5.5 (2.1)
Death within 1st year, n (%)	7 (21.8)
Lost to follow-up, n (%)	5 (15.6)
Patients with min. Follow-up, n (%)	20 (62.5)
Disease causing pathogens^a^	
S. aureus, n (%)	11 (34.4)
S. epidermidis, n (%)	9 (28.1)
Others, n (%)	12 (37.5)
Level of AKA	
Proximal, n (%)	5 (15.6)
Midshaft, n (%)	11 (34.4)
Distal, n (%)	16 (50.0)
Fitted with functional prosthesis^b^, n (%)	16 (80.0)
Mechanic knee joint, n (%)	6 (30.0)
Microprocessor knee joint, n (%)	10 (50.0)

^a^Disease causing pathogen isolated in PJI leading to AKA

^b^Out of 20 patients, which were available for FUP

### Comparison of complications and functional outcome of MKA and AKA

Patients with AKA showed a tendency towards a higher postoperative death rate with 13% when compared with patients with MKA (4%) (*p* = 0.081). Major complications, which required surgical revision, were seen in both cohorts equally, in 36% after MKA (*n* = 20) and in 35% after AKA (*n* = 7) (*p* = 0.91). Recurrence of in infection was the most common complication and occurred within follow-up interval in 22% after MKA (*n* = 12) and in 35% after AKA (n = 7) (*p* = 0.25). Due to this, amputation had to be performed in six cases after MKA (11%) and re-amputation was necessary in four (20%) after AKA (*p* = 0.31).

The functional outcome and quality of life, which were assessed in average 53 months after MKA and 62 months after AKA showed now significant differences between both procedures. Patients after amputation reached an average LEFS of 37 points and a mean PCS of 36, whereas after arthrodesis a mean LEFS of 28 points (*p* = 0.181) and a PCS of 30 (*p* = 0.080) could be observed. In both cohorts a comparable mental quality of life could be observed with a mean MCS for AKA and MKA of 47 and 46, respectively (*p* = 0.755) (Table 2). In total ten AKA patients could be fitted with a modern microprocessor-controlled-knee-joint. This sub-group showed a significantly better functional outcome with a mean LEFS of 56, compared to patients with a mechanic knee joint (mean LEFS: 20, *p* < 0.01) or those who received an arthrodesis (*p* < 0.01). Four patients that couldn’t be fitted with prosthesis had with a mean LEFS of 14 a significantly compromised outcome when compared with MKA patients (*p* < 0.01).

In amputee patients age at surgical amputation was associated with a significantly lower functional outcome and quality of life at final follow-up examination (*p* < 0.01). Patients aged less than 60 years could all be fitted with prosthesis and reached a mean LEFS of 56, patients aged 60 to 69 years showed a mean LEFS of 36 and those who were aged between 70 and 79 years had a mean LEFS of just 14. All patients aged older than 80 years at amputation were lost to follow-up. In contrast, in patients receiving arthrodesis age did not significantly influence the functional outcome, since the age groups of below 60 years, 60 to 69 years, 70 to 79 years and more than 80 years showed a comparable LEFS value of 28, 30, 28 and 27 respectively.

## Discussion

If infection after septic failure of TKA cannot be controlled or multiple revisions led to extensive bone or soft-tissue damage, salvage of a failed TKA remains difficult and the only alternatives to regain mobility and quality of life for the patient are AKA or KA [7, 10, 18–20]. Previous research does not provide a proper answer, if knee-arthrodesis or AKA with proper orthotic care is superior in terms of functional outcome, quality of life and postoperative complications. Therefore we compared these parameters in patients with AKA and knee-arthrodesis after PJI and revealed that as well AKA as knee-arthrodesis patients suffered an equally high rate of major complications of around 35%. Correspondingly, patients with AKA and knee-arthrodesis after septic failure of revision TKA showed a comparably compromised functional outcome and physical quality of life, whereas AKA patients that were fitted with a microprocessor-controlled-knee-joint reached a significantly better functional outcome, compared to all other amputee patients or those who received arthrodesis. Therefore, patients with a proper physical and mental state that will be able to mobilize with proper orthotics may benefit from an AKA. In amputee patients increasing age was associated with a lower functional outcome and decreasing number of patients fitted with prosthesis. In contrast, in arthrodesis patients age did not influence the functional outcome.

In literature a comparable complication rate of 31–32% after AKA [10, 21] and 30–50% after knee-arthrodesis is reported [2, 11]. Recurrence of infection was the most common complication in our study populations, which surprisingly occurred with 35% more frequently after AKA, than after MKA (22%). It is astonishing that reinfection is less common after MKA, despite a huge implant is present. Infection in MKA led in 15% to implant loosening and in 50% of these cases no re-arthrodesis was possible and amputation had to be performed. The implant survival rate of MKA was after one year 86%, after five years 71% and after ten years 61%. These results are considerably higher than literature data, which showed survivorship of MKA of 50% and 25% at five and ten years, respectively [12].

Death, which was related to the underlying disease or a serious postoperative complication, occurred in 4% after MKA and in 13% after AKA. This and the higher re-infection rate may be explained that patients with AKA had a more compromised overall health status and that the underlying infection was more often caused by a more virulent pathogen, such as *S. aureus*. It is widely accepted that in uncontrolled and occasionally life-threatening infections amputation is the preferred treatment option.

In literature on knee-arthrodesis after PJI, contrary results about functional outcome and quality of life as well as high complication rates are reported [2, 7, 11, 18, 22]. It has to be noted that in several above-cited studies knee-arthrodesis was performed with bone fusion using external fixator or intramedullary nail. A specific problem of multiple revision TKAs is an increasing bone defect and therefore a direct bony fusion would result in a leg length discrepancy of more than 5 cm. Such a leg length discrepancy, is known as a major factor to reduce the functional outcome and quality of life [5, 7]. In our cohort we performed arthrodesis with modular endoprostheses. This technique provides above-mentioned advantages over bone fusion and allows the reconstruction of segmental deficits and consequently adaption of a leg length discrepancy [12, 23].

Conway et al. concluded in a literature review on knee-arthrodesis, that a patient with successful knee-arthrodesis may be able to walk effectively, particularly in comparison to AKA [18]. Further studies reported a very poor functional outcome after amputation above septic failure of TKA [8–10]. But, the listed studies and the studies cited by Conway et al. analysed amputations which were mainly performed in the 1970’s until the 1990’s. Functional results of this era are meanwhile obsolete and can’t be compared with nowadays. Meanwhile considerable engineering process with development of microprocessor-controlled knee-joints improved functional outcome and quality of life after AKA [24]. Our AKA cohort was fitted in 80% with prosthesis and mainly with a microprocessor-controlled -knee-joint, which may explain the deviating functional results compared to previous studies. In contrast, in the study of Sierra et al., who reported a poor functional outcome for AKA, just 36% of the patients were fitted with prosthesis [10]. Chen et al. stated a worse functional outcome for AKA when comparing with knee-arthrodesis after PJI. But in their AKA cohort also just 30% were fitted with prosthesis and no details are provided on the type of prosthesis.

The good functional outcome of our AKA population may also be explained by the fact that they showed a lower mean age with 63 years when compared to the MKA cohort (69 years). The age at amputation is significantly influencing the later functional outcome, as proven by our results.

The major limitation of the current study is that the patients are not prospectively randomized to one cohort. However, a randomization is ethically not acceptable. The decision process is depended on a multitude of factors such as soft tissue and bony situation, infection parameters, overall medical condition and the patients` preference. Nevertheless, the knowledge of the prognosis and what the individual patient has to expect from a MKA or an AKA in terms of quality of life or complication rates are important aspects in this decision process. Both cohorts were not matched in terms of age and gender, because AKA and MKA are rare procedures and matched cohorts with a representative sample-size are only feasible in a multi-center study. Further limitation is a lack of the pre-operative documentation of functional status and quality of life and ta functional outcome score. However, a pre-operative functional status has a limited value, because at this stage most of the patients are bedridden due to the underlying PJI. Furthermore the aim of the study was to compare the functional outcome between the two surgical procedures and not within one cohort. Another limitations are the inhomogeneous follow-up intervals and the high lost to follow-up rate of 32% after MKA and 37% after AKA. The inhomogeneous follow-up intervals are a consequence of the retrospective study design. The high drop out rate is explained by the advanced age of a part of patients at inclusion and is consequently accounting for a limited number of patients available for follow-up examination. At follow-up, the mean LEFS was 37 for AKA patients and 28 for MKA patients, but statistical analysis could not show any significant difference. The missing significance may be explained by the limited power of the study. Nevertheless, the current data provide basic information for a proper sample size calculation for a multicentre study. A multicentre study is needed for more reliable outcome data and the indications for AKA or MKA after septic failure of revision arthroplasty are rare and drop out rates in this cohort are high.

## Conclusion

Patients treated with AKA and MKA after septic failure of revision TKA showed a comparable functional outcome, quality of life and postoperative complication rate. Younger amputee patients, that could be fitted with microprocessor-controlled-knee-joint presented a significantly better functional outcome than MKA patients. If AKA patients could not be fitted with a prosthesis functional outcome was devastating. In unsalvageable situations of septic failure after TKA the treatment decision process is depending on the patients’ expectations, overall medical condition, physical strength, severity of infection and soft-tissue envelope. Taking these factors into account each case has to be evaluated carefully to determine which treatment option might lead to the best achievable outcome. For the daily clinical routine these data should be considered in the decision-making amputation vs. modular arthrodesis: Younger patients in a proper physical and mental state may benefit from an AKA with proper orthotics, whereas in physically compromised older patients arthrodesis seems to be the superior treatment.

## Abbreviations

AKA: Above the knee amputation
CCI: Charlson comorbidity index
LEFS: Lower Extremity Functional Scale
MCS: Mental component summary
MKA: Modular knee prosthesis
PCS: Physical component summary
PJI: Prosthetic joint infection
TKA: Total knee arthroplasty

## References

[1] Matar WY, Jafari SM, Restrepo C, Austin M, Purtill JJ, Parvizi J (2010). Preventing infection in total joint arthroplasty. The Journal of bone and joint surgery American volume.

[2] Rohner E, Windisch C, Nuetzmann K, Rau M, Arnhold M, Matziolis G (2015). Unsatisfactory outcome of arthrodesis performed after septic failure of revision total knee arthroplasty. The Journal of bone and joint surgery American volume.

[3] Mittal Y, Fehring TK, Hanssen A, Marculescu C, Odum SM, Osmon D (2007). Two-stage reimplantation for periprosthetic knee infection involving resistant organisms. The Journal of bone and joint surgery American volume.

[4] Mortazavi SM, Vegari D, Ho A, Zmistowski B, Parvizi J (2011). Two-stage exchange arthroplasty for infected total knee arthroplasty: predictors of failure. Clin Orthop Relat Res.

[5] Jones RE, Russell RD, Huo MH (2012). Alternatives to revision total knee arthroplasty. The Journal of bone and joint surgery British volume.

[6] Gottfriedsen TB, Schroder HM, Odgaard A (2016). Knee arthrodesis after failure of knee Arthroplasty: a Nationwide register-based study. The Journal of bone and joint surgery American volume.

[7] CH W, Gray CF, Lee GC (2014). Arthrodesis should be strongly considered after failed two-stage reimplantation TKA. Clin Orthop Relat Res.

[8] Isiklar ZU, Landon GC, Tullos HS (1994). Amputation after failed total knee arthroplasty. Clin Orthop Relat Res.

[9] Pring DJ, Marks L, Angel JC (1988). Mobility after amputation for failed knee replacement. The Journal of bone and joint surgery British volume.

[10] Sierra RJ, Trousdale RT, Pagnano MW (2003). Above-the-knee amputation after a total knee replacement: prevalence, etiology, and functional outcome. J Bone Joint Surg Am.

[11] Chen AF, Kinback NC, Heyl AE, McClain EJ, Klatt BA (2012). Better function for fusions versus above-the-knee amputations for recurrent periprosthetic knee infection. Clin Orthop Relat Res.

[12] Angelini A, Henderson E, Trovarelli G, Ruggieri P (2013). Is there a role for knee arthrodesis with modular endoprostheses for tumor and revision of failed endoprostheses?. Clin Orthop Relat Res.

[13] Della Valle C, Parvizi J, Bauer TW, DiCesare PE, Evans RP, Segreti J, Spangehl M, Watters WC, 3rd, Keith M, Turkelson CM et al: American Academy of Orthopaedic surgeons clinical practice guideline on: the diagnosis of periprosthetic joint infections of the hip and knee. The Journal of bone and joint surgery American volume 2011, 93(14):1355–1357. 10.2106/JBJS.9314ebo 21792503

[14] Charlson ME, Pompei P, Ales KL, MacKenzie CR (1987). A new method of classifying prognostic comorbidity in longitudinal studies: development and validation. J Chronic Dis.

[15] Binkley JM, Stratford PW, Lott SA, Riddle DL (1999). The lower extremity functional scale (LEFS): scale development, measurement properties, and clinical application. North American Orthopaedic rehabilitation research network. Phys Ther.

[16] Ware J, Kosinski M, Keller SD (1996). A 12-item short-form health survey: construction of scales and preliminary tests of reliability and validity. Med Care.

[17] Jenkinson C, Layte R, Jenkinson D, Lawrence K, Petersen S, Paice C, Stradling J (1997). A shorter form health survey: can the SF-12 replicate results from the SF-36 in longitudinal studies?. J Public Health Med.

[18] Conway JD, Mont MA, Bezwada HP (2004). Arthrodesis of the knee. J Bone Joint Surg Am.

[19] Rao N, Crossett LS, Sinha RK, Le Frock JL (2003). Long-term suppression of infection in total joint arthroplasty. Clin Orthop Relat Res.

[20] Thornhill TS, Dalziel RW, Sledge CB (1982). Alternatives to arthrodesis for the failed total knee arthroplasty. Clin Orthop Relat Res.

[21] Fedorka CJ, Chen AF, McGarry WM, Parvizi J, Klatt BA (2011). Functional ability after above-the-knee amputation for infected total knee arthroplasty. Clin Orthop Relat Res.

[22] Bargiotas K, Wohlrab D, Sewecke JJ, Lavinge G, Demeo PJ, Sotereanos NG (2006). Arthrodesis of the knee with a long intramedullary nail following the failure of a total knee arthroplasty as the result of infection. The Journal of bone and joint surgery American volume.

[23] Somayaji HS, Tsaggerides P, Ware HE, Dowd GS (2008). Knee arthrodesis--a review. Knee.

[24] Bellmann M, Schmalz T, Blumentritt S (2010). Comparative biomechanical analysis of current microprocessor-controlled prosthetic knee joints. Arch Phys Med Rehabil.

